# Supplementary material for: Affinity enrichment of extracellular vesicles from plasma reveals mRNA changes associated with acute ischemic stroke
Source: Commun Biol. 2020 Oct 26;3:613. doi: 10.1038/s42003-020-01336-y (PMC7589468; doi:10.1038/s42003-020-01336-y)
Supplement: Supplementary file 1 — Supplementary information [file 42003_2020_1336_MOESM1_ESM.docx]

**Supplementary Information**

**Affinity enrichment of extracellular vesicles from plasma reveals mRNA changes associated with acute ischemic stroke**

**Supplementary Figures**

**
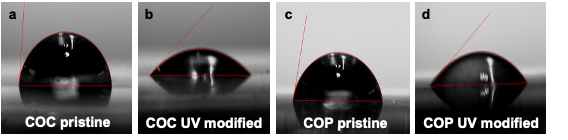
**

**Supplementary Figure 1.** Contact angle variation; (a) COC pristine, (b) COC UV/O_3_ modified, (c) COP pristine, (d) COP UV/O_3_ activated. (b) Water contact angle values of COC and COP thermoplastics before and after UV/O_3_ activation.

**
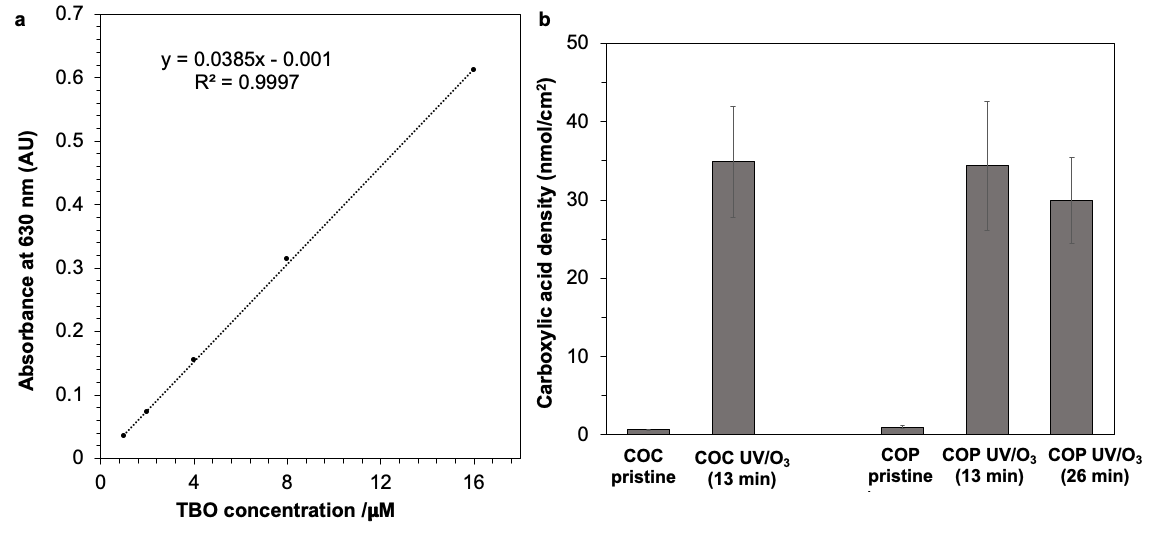
**

**Supplementary Figure 2.** (a) Calibration curve for TBO in 40% acetic acid measured at TBO’s absorption maximum, which is 630 nm. (b) Carboxylic acid surface densities calculated from the TBO assay for COC and COP thermoplastics.

**Supplementary Figure 3.** EVs diffused axially amidst Poiseuille flow, and their axial position was tracked with respect to the channel’s width (*W*). Simulations agreed with the analytical solution to Fick’s Second Law (hashed bars) when affinity-binding was disregarded in the Monte Carlo model (grey bars). When MAP affinity-binding was enacted (blue bars), the EV spatial distribution changes significantly, even within the channel’s width, because Fick’s law becomes invalid as EV concentration is no longer constant. Note that D = 5 µm^2^/s, L = 2.5 mm, W = 10 µm, V_ave_ = 1 mm/s, and Chang-Hammer dynamics were neglected so that all EV-Ab interactions were successful.


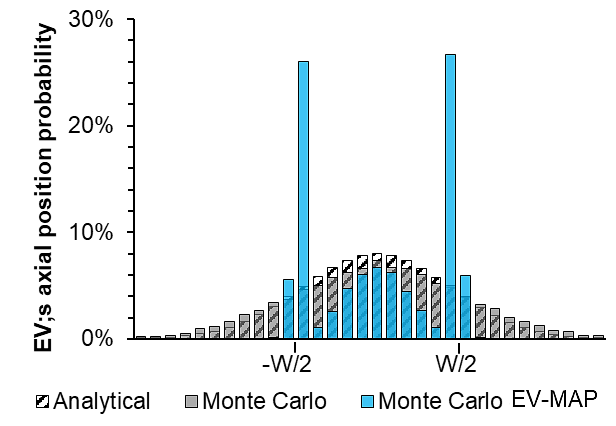


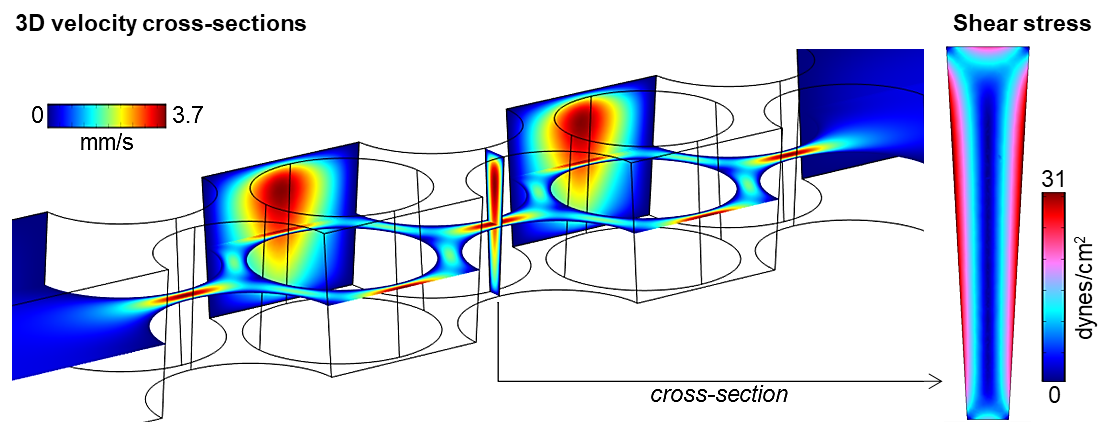


**Supplementary Figure 4.** COMSOL Multiphysics simulation results showing 3-dimensional velocity cross-sections and shear stress profiles between two adjacent micropillars. Note that symmetry constraints were placed along two sides of the simulation to achieve high element quality (**Supplementary Table 3**) at a tractable computational cost.


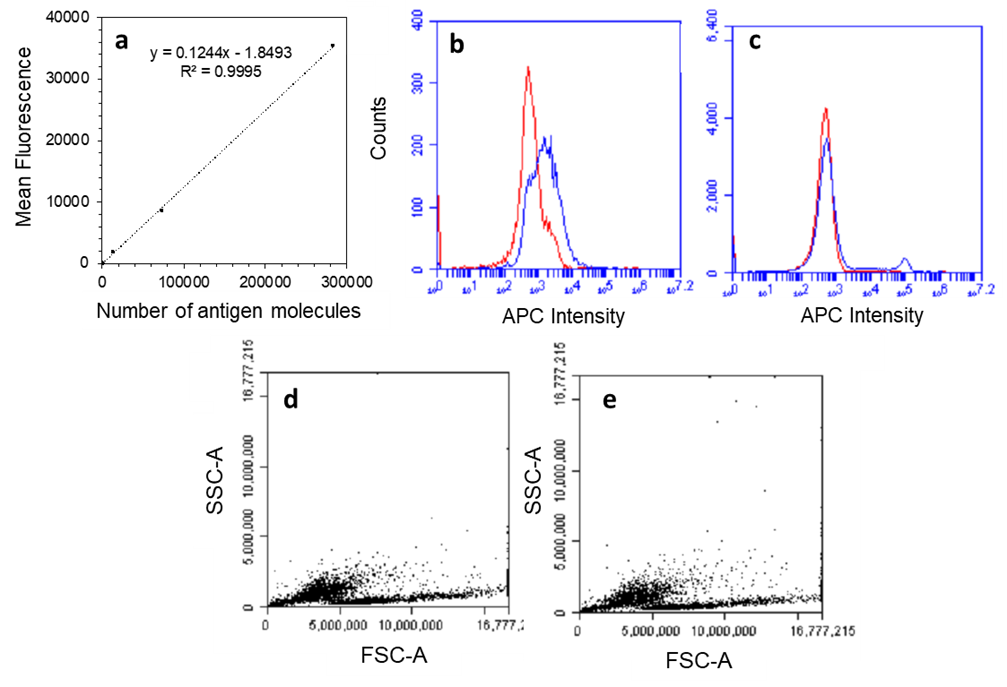


**Supplementary Figure 6.** Cells released from the sinusoidal cell isolation microfluidic device after staining with (a) DAPI, (b) anti-human CD45-FITC antibody, and (c) anti-human CD8α APC mAb.


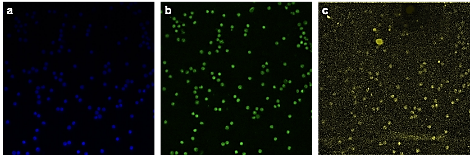


**Supplementary Figure 5.** (a) Calibration curve generated with ABC calibration beads. (b) Representative fluorescence histogram data for MOLT-3 cells stained with: red trace – APC conjugated IgG2B isotype; black trace – APC conjugated CD8 α mAb. (c) Representative fluorescence histogram data for buffy coat isolated from blood stained with: red trace – APC conjugated IgG2B isotype; blue trace – APC conjugated CD8α mAb. (d) FSC-A versus SSC-A dot plot for cells stained with APC conjugated IgG2B isotype. (e) FSC-A versus SSC-A dot plot for cells stained with APC conjugated CD8α mAb.


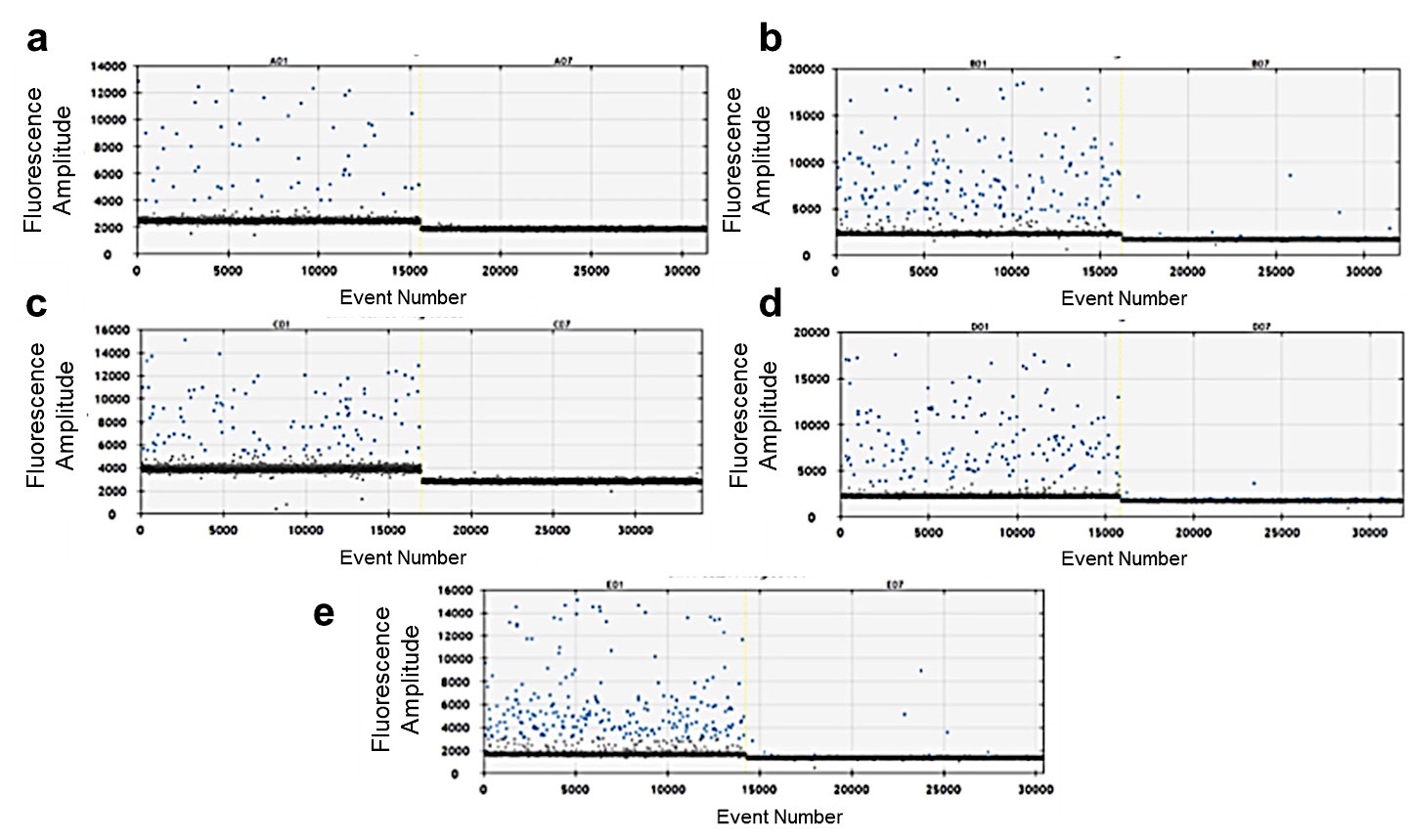


**Supplementary Figure 7**. Representative data from droplet ddPCR for MOLT-3 cell line; Left section: Data from positive RT reaction, Right section: Data from negative RT reaction. (a) PLBD1. (b) vFOS. (c) MMP-9. (d) CA4. (e) VCAN. 0.7 ng of TRNA was used for RT(+) and RT(-). cDNA was diluted 2.5x and used in ddPCR.

**Supplementary Tables**

**Supplementary Table 1.** Water contact angle variation for COC and COP thermoplastics.

| Thermoplastic | Contact Angle/^o^ | |
| --- | --- | --- |
|  | Pristine (n = 5) | UV/O_3_ modified (22 mV/cm^2^ , 13 min) (n = 5) |
| COC | 85.6 ± 2.5 | 33.7 ± 3.2 |
| COP | 79.7 ± 1.4 | 35.6 ± 3.9 |

**Supplementary Table 2.** Parameters used for Chang-Hammer dynamics (Eqs. S4-S8) in Monte Carlo simulation. CD8 receptor surface densities were calculated via antigen expression results by flow cytometry and assuming a cell diameter of 8 μm.

| Property | Value | Reference |
| --- | --- | --- |
| EV diameter | 50-150 nm | - |
| Diffusion coefficient (D) in plasma | 5-15 μm^2^/s | - |
| CD8 surface density (C_∞_) | 12.3 µm^-2^ | - |
| Antibody binding kinetics (k_in_) | 2.5 M^-1^ s^-1^ | 6 |
| Ab-antigen encounter radius (a_i_) | 2 nm | ^5,7^ |

**Supplementary Table 3.** Parameters used for 3-dimensional COMSOL simulations of laminar flow dynamics in the 3-bed device prototype. Geometry parameters were derived from confocal, laser-scanning profilometry measurements.

| Parameter | Value |
| --- | --- |
| Physics | Laminar flow, stationary |
| Viscosity | 0.0345 Pa s |
| Density | 1.055 g/mL |
| Inlet | 2.1 µL/min (0.21 mm/s) |
| Outlet | 0 Pa |
| Pillar height | 90 µm |
| Pillar diameter | 110-120 µm |
| Inter-pillar spacing | 10-20 µm |
| Draft angle | 4.6° |
| Program | COMSOL 5.2a |
| Number of elements | 11,528,872 |
| Average element quality | 0.6754 |
| Solver | GMRES |
| Tolerance | 10^-3^ |

**Supplementary Table 4.** Primer sequences used in gene expression analysis.

| Gene | Primer F 5’-3’ (Tm / °C) | Primer R 5’-3’ (Tm / °C ) | Amplicon size (bp) | Span from polyA (nt) |
| --- | --- | --- | --- | --- |
| *vFOS* cDNA clone MGC:11074 IMAGE:3688670 | TGCCAGGAACACAGTAG  (51.4) | TTCAGAGAGCTGGTAGTTAG (50.7) | 188 | 301 |
| *VCAN*  cDNA clone IMAGE:5218077 | TCTCAAAGAAACAGAGTGATA  (49.9) | AGAGCCACAGAGCATTT  (51.1) | 156 | 390 |
| *PLBD1*  NM_024829.5 | GTACTGAGATGCTAGGTAGATA (50.2) | CAAGGGAAAGTGACTGATAC (50.4) | 189 | 470 |
| *MMP-9*  NM_004994.2 | GGGATTTACATGGCACTG  (50.8) | ACCGAGAGAAAGCCTATT  (50.2) | 162 | 370 |
| *CA4* cDNA clone MGC:71638IMAGE:30331755 | GAAGCCTGGAACTTGGA  (51.7) | AGCGCACGGTGATAAA  (51.4) | 164 | 240 |

**Supplementary Table 5**. Representative data from droplet digital PCR (clinical sample and MOLT-3 cell line).

| Gene | Sample data from a clinical sample | | | | Sample data from MOLT-3 cell line sample | | | |
| --- | --- | --- | --- | --- | --- | --- | --- | --- |
|  | Copies per  20 µL | Accepted Droplets | Negative Copies per  20 µL | Accepted  Droplets | Copies per  20 µL | Accepted Droplets | Negative Copies per  20 µL | Accepted  Droplets |
| *PLBD1* | 36 | 14725 | 7.6 | 12297 | 94 | 15637 | 0 | 15717 |
| *vFOS* | 130 | 14612 | 4 | 12043 | 272 | 16272 | 4.4 | 15716 |
| *MMP9* | 112 | 15310 | 9 | 12949 | 168 | 17055 | 0 | 16976 |
| *CA4* | 104 | 14774 | 4 | 12043 | 248 | 15866 | 1.4 | 15992 |
| *VCAN* | 340 | 14877 | 13 | 12707 | 456 | 14291 | 5.8 | 16114 |

**Supplementary Table 6.** Patient information.

| Single blinded patient code # | Patient age/gender | Diagnosis | Blood draw date |
| --- | --- | --- | --- |
| 1 | 59/F | Control | 3/10/2011 |
| 3 | 61/F | Control | 6/3/2011 |
| 5 | 83/F | Control | 5/27/2011 |
| 7 | 61/M | Control | 6/15/2011 |
| 9 | 53/M | Control | 7/19/2011 |
| 2 | 66/M | AIS patient | 7/22/2011 |
| 4 | 72/M | AIS patient | 8/4/2011 |
| 6 | 67/M | AIS patient | 8/18/2011 |
| 8 | 95/F | AIS patient | 11/7/2011 |
| 10 | 85/F | AIS patient | 12/12/2011 |

F-female, M-male

**Supplementary Table 7**. TRNA yields isolated from affinity selected EVs from clinical samples.

| Single blinded  patient code # | RNA isolated (ng) | Mass (ng) of RNA per RT (+/-) reaction |
| --- | --- | --- |
| 1 | 1.79 | 0.48 |
| 2 | 2.16 | 0.50 |
| 3 | 1.38 | 0.35 |
| 4 | 4.44 | 1.07 |
| 5 | 2.22 | 0.50 |
| 6 | 0.54 | 0.15 |
| 7 | 0.60 | 0.14 |
| 8 | 0.44 | 0.11 |
| 9 | 0.43 | 0.11 |
| 10 | 0.64 | 0.15 |

Note: 0.7 ng and 1.1 ng of TRNA isolated from MOLT-3 and clinical sample, respectively was used in RT(+) and RT(-). cDNA was diluted 2.5x and used in ddPCR.

**Supplementary Methods**

***Expression of CD8 antigens in T-cells and MOLT-3 cell line***

MOLT-3 cells were centrifuged at 300 g for 10 min. The cells were then resuspended in 0.5% (w/v) bovine serum albumin in PBS. The cell count in the sample as evaluated by flow cytometry was 5.0 x 10^5^ cells/mL. Next, the sample was divided into aliquots (200 µL) and 10 μL of APC conjugated mouse IgG2B anti-hCD8α mAb (mono-clonal antibody) was added to one aliquot and 10 μL APC conjugated mouse IgG2B isotype control to the other portion.

Three mL of a blood sample was layered on top of equal volumes of Histopaque 1077 (Sigma-Aldrich) without mixing and the solution was centrifuged at 400 g for 30 min. The buffy coat was carefully aspirated and was then centrifuged at 300 g for 10 min and the pellet resuspended in PBS, washed, and centrifuged again with the washing repeated 3x. The cell pellet was resuspended in 400 µL of 0.5% (w/v) BSA in PBS. Staining was the same as for the MOLT-3 cell line.

ABC beads (Bangs Laboratories, Inc, Quantum Simply Cellular anti-mouse IgG, with antibody binding range between 9,533 and 528,475) were incubated with mouse anti-human CD8α antibodies (same concentration as for cells). Beads and cell samples were analyzed using a BD Accuri C6 plus flow cytometer (10,000 data points collected for beads and MOLT-3). Gating was set from T-cells with 20,000 events. The data were analyzed with software provided by the manufacturer.

***Comparison of Cyclic Olefin Copolymer (COC) and Cyclic Olefin Polymer (COP) thermoplastics***

Polymer plaques that were used in these studies included COP (Zeonor 1060R) and COC (Topas, 6013s-04). Polymer plaques were dried in an oven at 65°C overnight. Contact angle and TBO assays were then performed on both COC and COP plaques.

***Water contact angle measurements***

Small pieces (2 cm x 2 cm) of COC and COP thermoplastics were cleaned by sonicating in 10% Micro-90 for 5 min and then rinsed with IPA and nanopure water. The thermoplastics were dried at 70°C overnight before measuring the water contact angle. Both pristine and UV-modified COC and COP were used for contact angle measurements, which were performed using a VCA Optima instrument (AST products). Two μL of water (18.2 MΩ.cm at 25°C) was dispensed onto the thermoplastic surfaces and a photograph of each droplet captured immediately for analysis using the software provided by the manufacturer. The measurements were repeated five times at different positions.

***Carboxylic acid density measurements***

COC and COP used for fabrication of the EV-MAP devices were characterized following photoactivation by UV/O_3_ that generates surface-confined carboxylic acid scaffolds for mAb surface attachment. Carboxylic acid surface densities of COC and COP thermoplastics were measured as reported previously.^1^ In brief, the substrate’s surface was placed in an *in situ* incubation chamber (Bio Rad) filled with 0.1% (w/v) Toluidine Blue O (TBO) (Thermo Fisher Scientific) in carbonate buffer (50 mM, pH =10.5). After 15 min, the substrate was submerged in the same buffer for 15 min and then air dried. This step removed any TBO molecules that were non-specifically attached to the substrate’s surface. To desorb the TBO molecules attached to the carboxylic groups, a 40% acetic acid (d = 1.0196 g mL^-1^) wash was used. The volumes used for the desorption were collected into pre-weighed tubes and analyzed with a Ultrospec 4000 UV/Vis spectrophotometer (Pharmacia Biotech) against a 40% acetic acid blank at TBO’s absorption maximum, which is 630 nm. A calibration curve was generated using TBO standards in 40% acetic acid. For the calibration curve, known amounts of TBO were directly added to the buffer solution.

**Supplementary Discussion**

**COC and COP thermoplastics water contact angles**

The 7-bed devices used for isolation of EVs from clinical samples were made from COP while the 3-bed device was made from COC. Properties of COC and COP thermoplastics were evaluated using water contact angle measurements to determine the extent of –COOH group formation in each plastic. Wettability of surfaces was compared by measuring the water contact angles before and after UV/O_3_ activation (**Supplementary Fig.** **1a**, **b, c and d**).

Pristine COC and COP thermoplastics showed similar values in their water contact angles indicative of a hydrophobic surface. Upon UV/O_3_ treatment of the surface, formation of carboxylic acid groups among others are generated,^1^ and the thermoplastics became more hydrophilic with a subsequent drop in the water contact angle values (**Supplementary Fig.** **1a, b, c and d**). Calculated water contact angles for COP and COC thermoplastics are listed in **Supplementary Table 1**, which indicated that both of these thermoplastics have similar hydrophilicity after UV/O_3_ activation.

***Carboxylic acid densities on COC and COP surfaces***

A TBO assay was performed to determine the carboxylic acid surface density using a calibration curve generated by adding known amounts of TBO to a buffer solution (**Supplementary Fig.** **2a**). For both pristine COC and COP, TBO values indicated that a –COOH surface density of 0.69 ±0.06 nmol/cm^2^ and 0.97 ± 0.17 nmol/cm^2^, respectively, were generated following UV/O_3_ activation. After 13 min of UV/O_3_ activation, the calculated carboxylic acid surface densities were 34.88 ±7.10 nmol/cm^2^ and 34.39 ±8.21 nmol/cm^2^ for COC and COP, respectively. UV/O_3_ activation of the COP thermoplastic for an additional 13 min show no significant increase in -COOH group densities (**Supplementary Fig.** **2b**).

***Diffusion dynamics in the Monte Carlo simulations of the EV-MAP device***

The dynamics of EV affinity-selection can be split into two separate events: (1) Delivery of EVs from solution to the device’s surface where the capture antibodies are located; and (2) binding of the surface-bound Ab to the EV. The efficiencies of both processes dictate device recovery. We developed a Monte Carlo fluid dynamics simulation incorporating chemical physics and fluid dynamic principles to guide the design of micropillar-based devices. Previously, we outlined renditions of these chemical physics for CTC affinity-selection^2,3^ and diffusion models for the affinity-selection of labeled membrane proteins,^4^ although the diffusion model presented herein is a significant advancement compared to our previous reports.

As can be seen in **Figure f** of the main text, the inter-pillar space was modeled as a linear fluidic channel with a constant width and depth determined by the pillar spacing and pillar height, respectively. For diamond-shaped pillars, this spacing is constant and as such, the linear velocity when operated under a constant volume flow rate is the same irrespective of location in the pillared bed as noted in **Figure h**.

The delivery of EVs to the antibody-coated surface is limited by diffusion through the plasma matrix. As an EV is hydrodynamically transported through the device, it diffuses laterally and longitudinally according to Fick’s Second Law of diffusion. Over a small time increment, $\Delta t$, the probability that an EV will diffuse a distance $x_{D}$ from its initial position is given by a Gaussian distribution, $P(x)$:

$P\left( x \right)=\frac{1}{\sigma\sqrt{2\pi}}e^{-\frac{x_{D}^{2}}{2\sigma^{2}}}$ (Eq. S1)

This Gaussian distribution has a standard deviation given by$\sigma=\sqrt{2D\Delta t}$, where $D$ is the EV’s diffusion coefficient. Thus, smaller EVs with higher $D$ are more likely to diffuse further in the time interval $\Delta t$.

In addition to diffusive transfer, the EVs experience Poiseuille flow. In a high aspect ratio microchannel with a width of $W$, the EV’s forward velocity at position $x$ from the channel’s midline can be approximated by:

$V\left( x \right)=1.5 V_{ave}(1-\left( \frac{x}{W/2} \right)^{2}$ (Eq. S2)

In Eq. S2, $V_{ave}$ is simply calculated by dividing the volumetric flow rate by the channel’s cross-sectional area. The consequences of the parabolic flow profile in Eq. S2 are complex. As the EV diffuses closer to the surfaces, the EV forward motion slows, and more time is given for diffusion to occur. Consequently, the residence time of two EVs within the same device will not be the same if they take different random paths through the device.

We used a Monte Carlo approach to simulate the flow path of individual EVs through our pillared devices. This process was repeated until the average recovery converged to a constant value. This model allowed us to test various device bed lengths, inter-pillar spacings ($W$), and average flow velocities ($V_{ave}$) to design architectures with high recovery, high throughput, and high surface areas.

For each EV, the Monte Carlo simulation propagates an EV’s axial position ($x$ dimension) and longitudinal position ($y$ dimension) over finite time steps ($\Delta t$):

$x\left( t \right)=x\left( t-\Delta t \right)+rand(P(\sigma\left( D,\Delta t \right))$ (Eq. S3a)

$y\left( t \right)=y\left( t-\Delta t \right)+V\left( x\left( t-\Delta t \right) \right)+rand(P(\sigma\left( D,\Delta t \right))$ (Eq. S3b)

In Eq. S3a, the EV’s lateral $x$ position changes with axial diffusion over $\Delta t$ using $rand(P(\sigma\left( D,\Delta t \right))$, which is given by a pseudo-random number generator that moves the EV laterally according to a Gaussian $P(x)$ distribution with standard deviation $\sigma$. Longitudinal diffusion was considered in the same manner by Eq. S3b, but the EV had an additional term due to Poiseuille flow, namely the $V\left( x\left( t-\Delta t \right) \right)$ term described in Eq. S2.

***mAb-binding dynamics in the Monte Carlo simulations of EV-MAP recovery***

As an EV diffuses to and interacts with the device’s surfaces, successful binding between the surface-bound mAb and the transient EV is not guaranteed in a single encounter. In general, multiple encounters are necessary for successful EV/mAb binding. Herein, we adopted the Chang-Hammer model^5^ to describe this process.

The Chang-Hammer model describes the binding process between surface-confined mAbs and transient antigens, such as those present on the membrane of an EV. This model considers mAb-antigen binding kinetics, the transient motion of the antigen and its associated residence time in proximity to the surface-confined Ab, and the distance over which the EV rolls along the surface. Previously, we reduced the Chang-Hammer model to a few key equations,^2^ and herein, we adopted these dynamics.

First, as the EV rolls along the device’s surface, the forward rate constant $k_{o}$ for an encounter of antigens with a surface-confined mAb is:

$k_{o}=2a_{i}V_{eff}$ (Eq. S4)

In Eq. S4, $a_{i}$ is the mAb-antigen interaction radius (2 nm), and $V_{eff}$ is the velocity of the antigen relative to the surface, which is roughly half (0.47) the rolling EV’s velocity due to the opposing rotational motion produced by the EV’s surface. Furthermore, as the antigen encounters the mAb, the probability that they complex ($P$) is a function of both the mAb’s binding kinetics, $k_{in}$, and the encounter duration;

$P=\frac{k_{in}}{k_{in}+1/\tau}$ (Eq. S5)

$$\tau=8a_{i}/3\pi V_{eff}$$

As the EV’s linear velocity increases, $\tau$ decreases, yielding less time available for the mAb and antigen to complex, and $P$ decreases as well. Both the encounter rate, $k_{o}$, and the binding probability, $P$, are weighted against one another to yield an effective forward rate constant, $k_{f}$:

$k_{f}=k_{o}P$ (Eq. S6)

Lastly, the overall rate of EV adhesion $k_{ad}$ combines $k_{f}$ with the EV’s antigen surface density, $C_{\infty}$;

$k_{ad}=k_{f}C_{\infty}$ (Eq. S7)

To review, $k_{ad}$ considers the EV’s antigen expression and the velocity of the EV’s antigens, both in terms of how often the antigens encounter mAbs and how probable a binding event will occur given the balance of antigen-mAb interaction time and the mAb’s binding kinetics. To relate $k_{ad}$ to experimental parameters, consider an EV rolling along a mAb-coated surface at a linear velocity ($V$) for only a limited distance ($L$). The percent of EVs that will bind is:

${\%}_{bound}=1-{1/e}^{\frac{k_{ad}L}{V}}$ (Eq. S8)

Two aspects of Eq. S8 that improve EV recovery are immediately apparent: (i) Decrease the linear velocity; and (ii) maximize the interaction length between the EV and the surface. Unlike CTC dynamics,^2^ EVs have a relatively higher diffusion coefficient and the dynamics of an EV rolling along the micropillar surface are associated with a Peclet number <1. Very little can be done to counter lateral diffusion and control or manipulate the length of any given EV-micropillar interaction. Further, changing the bulk flow rate will do little to effect Eq. S8, which only describes the velocity at the surface, because surface flow velocities are limited to approximately zero by the no-slip condition. The velocity in Eq. S8 is more likely to be affected by EV diffusion rather than fluid velocity. Thus, the probability of mAb-binding is dictated by the binding dynamics of the affinity-agent and external manipulation of the device’s processing parameters (decreasing fluid velocity, decreasing inter-pillar spacing), which largely affect the diffusion-based delivery of EVs to the surface.

***Implementation of physical dynamics into Monte Carlo model and model validation***

The flow profile through the device bed with length $L_{bed}$ experienced by an EV was approximated as a straight microfluidic channel with a width $W$ equal to the interpillar spacing and length $L= L_{bed}C$, where $C$ is a correction factor linked to elongation of the flow path due to the pillar’s geometry. For diamond micropillars, $C=\sqrt{2}\approx1.41$, and for circular micropillars, $C=\pi/2 \approx1.57$.^4^

EVs were initiated at 11 positions along the pseudo-channel’s midline, and the EV’s position through the channel was propagated by using Eqs. S3a and S3b. If the EV encounters the channel’s surface ($x=\pm W/2$), the EV is propagated by multiplying $V(x)$ (Eq. S2) by the simulation’s time step $\Delta t$, and the probability of binding was calculated via Eqs. S4-S8. The binding probability was turned into an actionable decision (*i.e.,* binding or not) by using a pseudo-random number generator uniformly distributed between 0 and 1. If the random number was less than Eq. S8’s binding probability, the EV was recovered. If not, the EV’s position was propagated further via Eqs. S3a and S3b. This series of events continued until either the EV was recovered or the EV was lost ($y=L$).

Each EV’s track is a binary event, recovered or lost, and thousands of EVs were tracked until the simulated recovery converges, defined herein as a <0.01% change in average recovery when additional EVs were tracked. An additional convergence criterion stipulated a <10% standard deviation for five repetitive simulations. Lastly, given various $V_{ave}$ were tested, the program’s discretization of time into $\Delta t$ time steps was added as a final convergence criterion; after halving the $\Delta t$ increment, the averaged solution from five simulations must differ by <1% else the simulations would be repeated after halving $\Delta t$ again.

The accuracy of the Monte Carlo program was first tested by removing all recovery effects and letting EVs freely diffuse; the analytical model of Fick’s Second Law (Eq. S1) then becomes fundamentally valid. The results from the Monte Carlo simulation agreed well with a Gaussian function produced via Eq. S1 (**Figure 1f**). After enabling device recovery but without Chang-Hammer dynamics, where any surface interaction was considered successful, EVs accumulated along the channel for a total recovery of 64%. The Chang-Hammer dynamics (see **Supplementary Table 2** for relevant parameter values used) governing the probability of mAb-EV binding in Eqs. S4-S8 were then activated (axial distribution not shown) and the device recovery dropped substantially to 16% for this set of simulation parameters; inter-pillar spacing of 10 µm but short bed length of 2.5 mm and average velocity of 1 mm/s, which reduced the overall time available for axial diffusion.

Lastly, we compared the Monte Carlo model to our previous, less precise model for the set of experimental data in Battle, *et al*.^4^ Our previous simulation, which did not take into account Chang-Hammer dynamics and did not couple diffusion with fluid flow, generated 68% recovery for membrane proteins, while the Monte Carlo method predicted 75% recovery, which better approached the experimental values of 90 ±2%. Further improvements to the model, namely improving the Poiseuille approximation (Eq. S2 and **Supplementary Fig.** **3**) to better approximate the flow profile around a pillar would include reduced flow velocities between pillar rows and increase residence time available for diffusion and overall recovery in the model. All subsequent simulations used the parameters in **Supplementary Table 2** for evaluating device recovery.

**Shear stress in pillared device** **from COMSOL simulations**

Shear stress profiles were derived from COMSOL Multiphysics simulations using the parameters in **Supplementary Table 3**. The 3-dimensional laminar flow simulation results are shown in **Supplementary Fig.** **4**.

***Characterization of MOLT-3 cell line***

Expression of CD8 antigens in the MOLT-3 cell line was determined by flow cytometry. MOLT-3 cells showed CD8 expression at an average of 2,500 CD8 receptors/cell (**Supplementary Fig.** **5a** ) in 13% of the population, according to previous reports.^8^ For the isotype, the calculated value was 1,212 (**Supplementary Fig.** **5b**). We followed the same protocol for determining the number of receptors on T-cells isolated from blood via Histopaque 1077 density gradient centrifugation. In healthy blood, the mean CD8(+) T lymphocyte percentage was ~30.2%.^9^ Flow cytometry results showed ~6,600 CD8 receptors for T-cells and 511 for the isotype control (**Supplementary Fig.** **5c**).

***Phenotyping of T-cells***

Cells isolated with anti-CD8α mAb modified chips were released with USER enzyme and collected into a 96 well plate, where they were stained with APC labeled hCD8α Ab (Clone number 37006, R&D Systems) and FITC-labeled CD45 Ab (Clone number 2D1, R&D Systems). After 30 min of incubation with antibodies, cells were washed and centrifuged three times with PBS and treated with Triton-X100/DAPI. Cells were centrifuged again at 300 g for 10 min, and washed again before imaging using a 200M inverted microscope (Zeiss) equipped with a 10x objective (0.3 NA, Plan NeoFluar), XBO 75 Xe arc lamp, single band Cy5 and Cy3 filter set (Omega optical), Cascade:1K EM-CCD camera (Photometric), and MAC 5000 stage (Ludl Electronic Products), all of which were computer-controlled via Micro-Manager. Final images were background subtracted and analyzed using Image-J software.

Released CD8(+) T-cells stained with nuclear DAPI are shown in **Supplementary Fig.** **6a**. The cells we isolated also showed positive staining for anti-human CD45-FITC (leukocyte common antigen, **Supplementary Fig.** **6b**). To determine the purity of the isolated cells, we also stained cells with antihuman-CD8α -APC mAb. Among isolated cells, 81 ±11 % (n=4) DAPI positive cells showed positive CD8 staining (**Supplementary Fig.** **6c**).

***Droplet digital PCR (ddPCR)***

***Primer design for droplet digital PCR***

Primers for the 5 genes used in this study were custom designed and purchased from Integrated DNA Technologies. **Supplementary Table 4** shows forward and reverse primer sequences and their melting temperatures.

Gene copy numbers obtained from ddPCR were normalized to ng total RNA (TRNA). With each positive reverse transcription reaction (with enzyme), negative reverse transcription reaction (without reverse transcriptase enzyme) was performed and the data from a clinical sample and from the MOLT-3 cell line are shown in **Supplementary Table 5** and **Supplementary Fig.** **7**.

***Patient information***

Blood samples from the AIS patients were collected at the SUNY Down State Medical Center, New York, NY according to an approved Institutional Review Board procedure. Plasma was isolated from blood in a following way: blood was centrifuged at 300 x g for 10 minutes at RT. Plasma layer was carefully removed from the whole blood components at the bottom of the tube and placed in another tube. Isolated plasma was centrifuged again at 1,000 x g for 10 minutes. Plasma supernatant was collected without disturbing bottom tube small pellet/debris and aliquots prepared. Plasma samples were stored at -80°C until further analysis.

**References**

1 Jackson, J. M. *et al.* UV activation of polymeric high aspect ratio microstructures: ramifications in antibody surface loading for circulating tumor cell selection. *Lab on a Chip* **14**, 106-117 (2014).

2 Jackson, J. M., Witek, M. A. & Soper, S. A. Sinusoidal microchannels with high aspect ratios for CTC selection and analysis. *Circulating Tumor Cells: Isolation and Analysis*, 85-126 (2016).

3 Witek, M. A. *et al.* Discrete microfluidics for the isolation of circulating tumor cell subpopulations targeting fibroblast activation protein alpha and epithelial cell adhesion molecule. *NPJ precision oncology* **1**, 24 (2017).

4 Battle, K. N. *et al.* Solid-phase extraction and purification of membrane proteins using a UV-modified PMMA microfluidic bioaffinity microSPE device. *The Analyst* **139**, 1355-1363, doi:10.1039/c3an02400h (2014).

5 Chang, K.-C. & Hammer, D. A. The forward rate of binding of surface-tethered reactants: Effect of relative motion between two surfaces. *Biophys J* **76**, 1280-1292 (1999).

6 Gaster, R. S. *et al.* Quantification of protein interactions and solution transport using high-density GMR sensor arrays. *Nat Nanotechnol* **6**, 314-320, doi:10.1038/nnano.2011.45 (2011).

7 Springer, T. A. Adhesion receptors of the immune system. *Nature* **346**, 425-434, doi:10.1038/346425a0 (1990).

8 Bettger, W. J. & McKEEHAN, W. L. Mechanisms of cellular nutrition. *Physiological reviews* **66**, 1-35 (1986).

9 Afzal, N. *et al.* Percentage of CD4+ and CD8+ T-lymphocytes in blood of tuberculosis patients. *Journal of Ayub Medical College Abbottabad* **22**, 182-186 (2010).
