# Supplementary material for: Affinity enrichment of extracellular vesicles from plasma reveals mRNA changes associated with acute ischemic stroke
Source: Commun Biol. 2020 Oct 26;3:613. doi: 10.1038/s42003-020-01336-y (PMC7589468; doi:10.1038/s42003-020-01336-y)
Supplement: Supplementary file 3 — Description of Additional Supplementary Files [file 42003_2020_1336_MOESM3_ESM.pdf]

Description of Additional Supplementary Files

File Name: Supplementary Data 1
Description: Figure 1 f: Monte Carlo simulation results for the 3-bed EVMAP.

File Name: Supplementary Data 2
Description: Figure 1 g: Calculated sample processing time for 3-bed (5µL/min) and 7-bed devices (20 µL/min).

File Name: Supplementary Data 3
Description: Figure 1 h: Results of Monte Carlo simulation for the 7-bed EVMAP

File Name: Supplementary Data 4
Description: Figure 2 h: NTA results and TEM images showing the number of EVs released during first and second USER⍰ enzyme release.

File Name: Supplementary Data 5
Description: Figure 2k: Percentage of EVs released during first and second release with USER⍰ enzyme.

File Name: Supplementary Data 6
Description: Figure 3a: Cell line viability when cultured with different LPS concentrations in culture medium.

File Name: Supplementary Data 7
Description: Figure 3c: mRNA gene expression profiles for CD8+ EVs cells (0.5 and 0.7 ng of RNA for stimulated and unstimulated respectively used in RT reactions). Figure 3d: mRNA gene expression profiles for CD8+ Molt-3 cells (0.7 ng RNA for stimulated and unstimulated was used in RT reactions). Figure 3e: Correlation between mRNA copies found in stimulated and unstimulated Molt-3 cells and EVs. Figure 3f: Correlation between mRNA copies found in EVs and Molt-3 cells in stimulated and unstimulated conditions.

File Name: Supplementary Data 8
Description: Figure 4d: Correlation plot of particle concentration (presumably EVs) with RNA mass isolated from affinity selected CD8+ EVs.

File Name: Supplementary Data 9
Description: Figure 4e: Boxplots comparing the gene expression of CD8+Tcells and CD8+EVs isolated from healthy donor plasma for PLBD1 for cells (n=5) and EVs (n=6). Figure 4f: Boxplots comparing the gene expression of CD8+T-cells and CD8+EVs isolated from healthy donor plasma for vFOS for cells (n=5) and EVs (n=6). Figure 4g: Boxplots comparing the gene expression of CD8+T-cells and CD8+EVs isolated from healthy donor plasma for MMP9 for cells (n=5) and EVs (n=6). Figure 4h: Boxplots comparing the gene expression of CD8+T-cells and CD8+EVs isolated from healthy donor plasma for CA4 for cells (n=5) and EVs (n=6). Figure 4i: Boxplots comparing the gene expression of CD8+T-cells and CD8+EVs isolated from healthy donor plasma for VCAN for cells (n=5) and EVs (n=6).

File Name: Supplementary Data 10
Description: Figure 5a: NTA images of EVs isolated from clinical sample #4 by PEG precipitation and affinity selected with anti-CD8α mAb uisng the 7-bed EV-MAP.

File Name: Supplementary Data 11
Description: Figure 5e: Heat maps presenting the EV mRNA expression profiles for sample #4.

File Name: Supplementary Data 12
Description: Figure 5f: NTA results for selected Samples 1,4,6,and 8.

File Name: Supplementary Data 13
Description: Figure 5g: mRNA expression profiling for selected genes in clinical samples. Figure 5h: Heat map analysis of clinical samples (marked with numbers) and healthy donors (identified with letters). Figure 5i: Principal component analysis for clinical samples (identified with numbers) and healthy donors (identified with letters).
